# Exploring the effects of operational mode and microbial interactions on bacterial community assembly in a one-stage partial-nitritation anammox reactor using integrated multi-omics

**DOI:** 10.1186/s40168-019-0730-6

**Published:** 2019-08-28

**Authors:** Yulin Wang, Qigui Niu, Xu Zhang, Lei Liu, Yubo Wang, Yiqiang Chen, Mishty Negi, Daniel Figeys, Yu-You Li, Tong Zhang

**Affiliations:** 1 Environmental Microbiome Engineering and Biotechnology Laboratory, Department of Civil Engineering, The University of Hong Kong, Pokfulam Road, Hong Kong SAR, People’s Republic of China; 2 School of Environmental Science and Engineering, China–America CRC for Environment & Health, Shandong University, 72#Jimo Binhai Road, Qingdao, 266237 Shandong Province People’s Republic of China; 3 Ottawa Institute of Systems Biology and Department of Biochemistry, Microbiology and Immunology, Faculty of Medicine, University of Ottawa, Ottawa, ON Canada; 4 Department of Civil and Environmental Engineering, Graduate School of Engineering, Tohoku University, 6-6-06 Aoba, Aramaki, Aoba-ku, Sendai, 980-8579 Japan

**Keywords:** One-stage partial-nitritation anammox, Microbial community assembly, Nitrogen cycle, Auxotrophies, Multi-omics

## Abstract

**Background:**

The metabolic capacities of anammox bacteria and associated microbial community interactions in partial-nitritation anammox (PNA) reactors have received considerable attention for their crucial roles in energy-efficient nitrogen removal from wastewater. However, a comprehensive understanding of how abiotic and biotic factors shape bacterial community assembly in PNA reactors is not well reported.

**Results:**

Here, we used integrated multi-omics (i.e., high-throughput 16S rRNA gene, metagenomic, metatranscriptomic, and metaproteomic sequencing) to reveal how abiotic and biotic factors shape the bacterial community assembly in a lab-scale one-stage PNA reactor treating synthetic wastewater. Analysis results of amplicon sequences (16S rRNA gene) from a time-series revealed distinct relative abundance patterns of the key autotrophic bacteria, i.e., anammox bacteria and ammonia-oxidizing bacteria (AOB), and the associated heterotrophic populations in the seed sludge and the sludge at the new stable state after deterioration. Using shotgun metagenomic sequences of anammox sludge, we recovered 58 metagenome-assembled genomes (MAGs), including 3 MAGs of anammox bacteria and 3 MAGs of AOB. The integrated metagenomic, metatranscriptomic, and metaproteomic data revealed that nitrogen metabolism is the most active process in the studied PNA reactor. The abundant heterotrophs contribute to the reduction of nitrate to nitrite/ammonium for autotrophic bacteria (anammox bacteria and AOB). Genomic and transcriptomic data revealed that the preference for electron donors of the dominant heterotrophs in different bacterial assemblages (seed and new stable state) varied along with the shift in anammox bacteria that have different metabolic features in terms of EPS composition. Notably, the most abundant heterotrophic bacteria in the reactor were more auxotrophic than the less abundant heterotrophs, regarding the syntheses of amino acids and vitamins. In addition, one of the abundant bacteria observed in the bacterial community exhibited highly transcribed secretion systems (type VI).

**Conclusions:**

These findings provide the first insight that the bacterial communities in the PNA reactor are defined by not only abiotic factors (operating mode) but also metabolic interactions, such as nitrogen metabolism, exchange of electron donors, and auxotrophies.

**Electronic supplementary material:**

The online version of this article (10.1186/s40168-019-0730-6) contains supplementary material, which is available to authorized users.

## Background

The bacteria mediating anaerobic ammonium oxidation (anammox) can anaerobically oxidize ammonium using nitrite as oxidant to produce dinitrogen gas [1–4]. The discovery of the anammox process and its direct application in wastewater treatment plants provide an energy-efficient way of nitrogen removal from wastewater. By 2014, over 100 anammox processing plants had been implemented worldwide [5]. Given that nitrite levels in wastewater are generally insufficient for anammox bacteria to derive energy for growth, one-stage and two-stage partial nitritation/anammox (PNA) bioreactor systems have been developed to partially oxidize ammonium to nitrite. For the two-stage bioreactor, the ammonium is partially oxidized by ammonia-oxidizing bacteria (AOB) to nitrite in the first-stage aerated reactor, and the remaining ammonium and nitrite are further converted to nitrogen gas in the second anammox reactor [6–9]. Under oxygen-limited conditions, one-stage bioreactors, by contrast, harbor both aerobic AOB and anammox bacteria in one reactor [5, 10–12]. Due to the lower investment cost, approximately 88% of all full-scale operating installations are one-stage configurations [5].

The complex microbial community structures, life strategies, and interactions between taxa are of crucial importance for the stable removal of nitrogen from wastewater. A comprehensive understanding of the anammox community will therefore provide new insights into this process. Several previous studies reported the PNA microbial community in lab-scale [13, 14] and full-scale wastewater treatment systems [15, 16] using high-throughput 16S rRNA gene sequencing, although these could only provide some insights into the microbial community structure. With the development of sequencing technologies, more insights into anammox evolution and metabolism have been obtained using the genome-centric approach [3, 17–19]. Lawson et al. [20] studied the microbial community interactions in a lab-scale anammox reactor based on the integrated metagenomic and metatranscriptomic approach.

However, we know much less about how environmental conditions and microbial interactions shape the microbial community assembly in anammox systems. As reported, anammox bacteria have been found in a wide variety of habitats and show a distinct niche differentiation in natural and engineered systems [21, 22], which may be affected by environmental factors, such as dissolved oxygen (DO), temperature, salinity, nitrite, and ammonium concentrations [23–25]. These studies suggested that anammox bacteria might be selected by environmental filtering. In addition, the microbial community may be simultaneously affected by multiple processes, including biotic interactions (e.g., commensalism, amensalism, mutualism, and parasitism) [26, 27], dispersal limitation, and stochastic demographics [28].

To test our hypothesis that environmental filtering under different operational strategies and biotic interactions simultaneously shape the anammox microbial community assembly and the composition of dominant organisms, we set up a lab-scale one-stage PNA reactor. The PNA reactor was inoculated with enriched anammox sludge from a lab-scale continuous stirred-tank reactor (CSTR). At the start-up stage, the reactor encountered deterioration under a continuous mode. After adjusting the reactor from CSTR to a sequencing batch reactor (SBR), the performance of this PNA reactor recovered and achieved a stable nitrogen removal rate. Shifts in microbial community structures from the start-up period to the stable state were captured using the high-throughput 16S rRNA gene sequences and the newly retrieved metagenome-assembled genomes (MAGs) from metagenomes of anammox sludge samples. In addition, the microbial community interactions among these newly recovered MAGs were characterized by metagenomic and time-series metatranscriptomic data (8 time points in 3 independent reaction cycles, a total of 24 samples). Moreover, metaproteomic data were used to further identify the end products of gene expression and confirm the observed gene expression activities. The integrated multi-omics approach in the present study will shed light on the potential roles of operational mode and biotic interactions in shaping the microbial community assembly in a PNA reactor.

## Methods

### Reactor operation

A working volume of a 1.6-L lab-scale PNA reactor was inoculated with anammox biomass from Tohoku University (Civil and Environmental Engineering). The anammox sludge was enriched from a continuous one-stage PNA reactor (over 5 years) at low DO concentrations (0.1–0.8 mg/L) [29]. At the start-up stage, the studied PNA reactor was first operated with a continuous feeding strategy following the previous parameters [29] but encountered a measurable deterioration. We adjusted the CSTR mode to the SBR operating mode on day 40 under oxygen-deficient conditions to recover the nitrogen removal performance. DO in the reactor could not be detected by a DO meter (HI2004 edge, Hanna Instruments Inc., RI, USA) during both aerobic and anaerobic phases. The operational details are described in Additional file 1: (S1).

### Sampling, DNA and RNA extraction

**Fig. 1:**
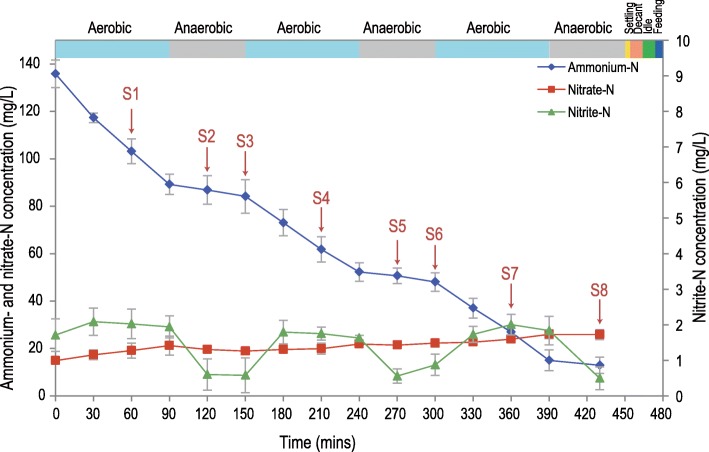
Variation of the nitrogen components over a reaction cycle and sampling points for the metatranscriptomic studies. The 8-hour cycle (480 min, 3 cycles/day) included 6 min feed phase, 450 min reaction phase (divided into 3 aerated and 3 non-aerated phases), 4 min settling phase, 10 min discharge phase, and 10 min idle phase. The replicates for DNA and RNA (S1–S8) extraction were collected from three independent reaction cycles on days 204, 210, and 213

To investigate the dynamics of microbial community structures, eight sludge samples (1.5 mL) were taken on days 1 (seed sludge), 19, 43, 118, 134, 156, 179, and 187 (Additional file 1: Figure S1), which covered the periods of start-up, deterioration, recovery, and new stable state. The suspended sludge samples were collected for 16S rRNA gene sequencing. DNA extraction of these 8 well-mixed sludge samples was performed using the FastDNA SPIN Kit for Soil (MP Biomedicals, France). Additionally, triplicate samples at 8 sampling points (24 sludge samples) were taken from 3 independent reaction cycles on days 204, 210, and 213 (Fig. 1), which were used for total DNA and RNA extraction and downstream genome recovery and transcription activity studies (Additional file 1: S2). The seed sludge (day 1) and one sludge sample that was taken on day 261 were used for shotgun sequencing to further check the microbial community composition.

### DNA and RNA sequencing

The 8 time-series DNA samples (days 1, 19, 43, 118, 134, 156, 179, and 187) that were used to study the changes in the microbial community structure were PCR-amplified with barcoded forward primer 515F and reverse primer 806R [30] and sequenced on the Illumina HiSeq 4000 platform (Illumina, CA, USA) to generate 250 bp paired-end reads (2 × 250 bp). The 3 DNA and 24 RNA samples (8 triplicate samples, 8 × 3) taken on days 204, 210, and 213 for the shotgun sequencing were sequenced on the Illumina HiSeq 4000 platform (Illumina, CA, USA) to generate 150 bp paired-end reads (2 × 250 bp) with 350 bp insert size (Additional file 1: S2). Total DNA that was extracted from the samples taken on days 1 and 261 was used for the shotgun sequencing as well.

### Protein extraction, trypsin digestion, and mass spectrometry analysis

Sludge samples were taken from a reaction cycle on day 261. The sampling points corresponded to S1, S3, S4, and S6 (Fig. 1). Total protein extraction was performed using the modified B-PER extraction method following the previous study [31, 32] (Additional file 1: S3). The trypsin digestion was performed following the previous study [33]. The tryptic digest was desalted with a 10-μm C18 column and dissolved in 0.1% formic acid (v/v) for analysis on a Q Exactive mass spectrometer (ThermoFisher Scientific Inc.) (Additional file 1: S3).

### DNA sequence processing

The clean 16S rRNA reads from the 8 sludge samples (normalized sequencing depth of 70,000) were obtained following the quality control pipeline Mothur MiSeq SOP [34]. QIIME (v 1.8.0) was then applied for open-reference operational taxonomic unit (OTU) picking at a cutoff of 98% [35]. UCLUST [36] was then applied to assign the OTU at a minimum similarity of 0.9 against the SILVA database (release 123) [37].

The clean metagenomic sequencing reads (average quality scores of > 30) generated from the 3 DNA samples (days 204, 210, and 213) were combined (461.5 million reads) for quality control. The clean reads were co-assembled using the CLC’s de novo assembly algorithm (CLC Genomics Workbench v6.04, CLCBio, Qiagen) with a *k*-mer of 35 and a minimum scaffold length of 1 kbp. A two-dimensional coverage binning approach was used to retrieve the MAGs of the microbial community members in anammox sludge [38] (Additional file 1: S4). The completeness of recovered MAGs was estimated using CheckM [39]. The relative abundance of MAGs was estimated based on the recruited reads of each MAG, which was normalized by the genome sizes. All annotated MAGs are available under the IMG system genome IDs listed in Additional file 2: Table S1.

### Metatranscriptomic analysis

The non-rRNA reads (ranging from 56 to 73 million) from 24 anammox sludge samples were mapped to all predicted ORFs of the co-assembled contigs using the read mapper of the CLC genomics workbench (v6.04, CLCBio, Qiagen) (Additional file 1: S4). Differential gene transcription across aerobic-anaerobic cycles was identified using the R package edgeR [40], at a *P* value cutoff of 0.001 for FDR and a fold change of 2. The non-rRNA reads transcripts per million measures (TPMs) were used as estimations of gene expression in the present study [41, 42]. To find the genes with relatively high transcription levels in each genome, the relative gene transcription was adopted in the present study [20], which relativized the TPM value of each ORF by the median TPM value calculated across the given genome. The overall gene transcription value of MAG was estimated based on the proportion of recruited metatranscriptomic reads of all ORFs of given MAG to all the reads that mapped to the ORFs of the recovered MAGs (Additional file 1: S4).

### Metaproteomic analysis

A metagenomic taxonomy-guided database search strategy was used for microbial protein identification [43]. The reconstructed database (Additional file 1: S5) search was performed using MetaPro-IQ approach as previously described [44]. Label-free quantification (LFQ) was performed using the implemented maxLFQ algorithm in MaxQuant [45, 46]. Proteins identified by the same set or a subset of peptides were grouped together as one protein group. Both razor and unique peptides were used for the protein quantification, and the minimum ratio count was set as 1. Taxonomic analysis of metaproteome data is described in detail in Additional file 1 (S5).

### Phylogenetic analysis and MAG annotation

A genome tree of the newly recovered MAGs and reference genomes was constructed using the Genome Tree DataBase (GTDB) with a concatenated set of 120 bacterial-specific conserved marker genes [47]. All retrieved MAGs were first uploaded to the Kyoto Encyclopedia of Genes and Genomes (KEGG) GhostKOALA [48] for the preliminary reconstruction of metabolic traits and were then uploaded to the Integrated Microbial Genomes Expert Review (IMG-ER) [49] system for genome annotation. The carbohydrate hydrolases were identified using HMMER [50] against the dbCAN database (September 2017) [51]. The peptidases were identified based on the BLASTP searches against the MEROPS (release 12.0) [52] (Additional file 1: S6). The subcellular location of the identified proteins was predicted using PSORT [53].

## Results and discussion

### Performance of the one-stage PNA reactor

The lab-scale one-stage PNA reactor was inoculated with anammox sludge from a continuous one-stage PNA reactor and followed the same continuous CSTR operating mode. Deterioration was observed during the first 75 days (Additional file 1: Figure S1, stage I). Although the same influent composition (concentration of ammonium was modified based on the amount of inoculated biomass), DO, pH, HRT, and temperature were adopted from the previous work [29]; the distinct bioreactor design and sludge mixing strategies (mixed using aeration in the previous work and stirred in the PNA reactor of this study) may change the performance of anammox sludge and induce deterioration at stage I. To recover the nitrogen removal performance, the continuously operating mode was changed to an SBR operating mode on day 40. After a 2-month recovery (stage II), the average nitrogen removal rate increased from 150.5 to 307.5 mgN/L/day and stayed at the new stable state (stage III). The color of the sludge (Additional file 1: Figure S2) changed from carmine (day 1) to peachpuff (day 85) and then gradually changed to blood-red (day 308). Self-aggregated granular anammox sludge was observed after approximately 150 days of operation and achieved an average granular size of 498 μm on day 308 (Additional file 1: Figure S3). This observed aggregation revealed that the microorganisms in the studied PNA reactor produced more extracellular polymeric substances (EPS) than those in the seed sludge. In the three reaction cycles for metagenomic and metatranscriptomic sequencing at stage III (days 204, 210, and 213), the ammonium decreased faster in the aerobic phases than in the anaerobic phases (Fig. 1). As the product of ammonium oxidation, nitrite did not obviously accumulate after a reaction cycle (~ 0.5 mg/L), further confirmed the high activity of anammox bacteria in the PNA reactor in a new stable state.

### Succession of bacterial community

**Fig. 2:**
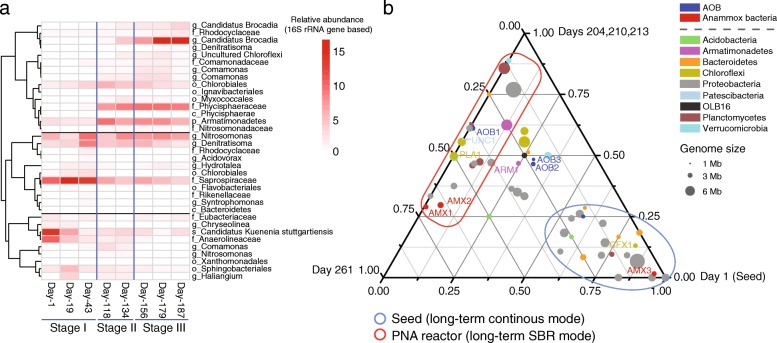
Dynamics in bacterial community assembly. **a** Heat map shows the dynamics of OTUs that have a relative abundance > 0.3% in at least half of the collected samples. Side dendrogram represents the hierarchical clustering based on Pearson correlation. **b** Ternary plot shows the abundance comparison of the 58 newly recovered MAGs in three different anammox sludge samples. The value of a given MAG in each sample is equal to its corresponding abundance divided by the abundance sum of this MAG in the three anammox sludge samples. The abundance of MAGs in the triplicate samples (days 204, 210, and 213) was averaged. The organisms that were mainly identified in the seed sludge and the studied PNA reactor are encircled with blue and red circles. The symbol size represents the genome size

The analysis of 16S rRNA gene sequences revealed that the microbial community underwent an obvious change (Additional file 1: Figure S4). It is noteworthy that the relative abundance of the phylum *Planctomycetes* showed a decreasing trend at stage I and then increased to as much as twofold more than that in the seed sludge at stage III. In addition, the dominant anammox population shifted from the genus *Candidatus* Kuenenia to *Candidatus* Brocadia (Additional file 1: Figure S4b). Regarding the relatively abundant OTUs that had a relative abundance > 0.3%, three clusters were obtained based on Pearson correlation (Fig. 2a). These patterns of bacterial communities, that is, increasing, decreasing, and transitionally increasing, further supported the shift of community composition in conjunction with changes in the operational mode. In these two different operational modes, nitrite-oxidizing bacteria (NOB) were nearly undetectable at an average relative abundance of 0.06%, suggesting that the operational modes effectively reduced the competition between anammox bacteria and NOB for nitrite.

After quality control, we obtained a total of 460,840,704 clean metagenomic reads from the samples on days 204, 210, and 213. Co-assembly of these reads generated a total of 104,581 scaffolds with an N50 of 12,180 bp, recruited 92.0% of total reads. Using the co-assembled metagenomes, we retrieved 49 MAGs, and the IDs of these MAGs were proposed based on their affiliated phyla [54] (Additional file 2: Table S1, Additional file 1: Figure S5). These newly recovered MAGs recruited 78.6% and 71.6% of the total DNA reads from the days 204, 210, and 213 samples and the sample from day 261, respectively, indicating that the overall community from day 261 was similar to the 3 days for genome binning. These MAGs from co-assembled metagenomes recruited 61.4% of the total DNA reads from day 1. Given the relatively low coverage of these MAGs in the seed sludge sample (day 1), individual assembly and binning were performed. We recovered 24 seed-MAGs (Additional file 2: Table S2) from the seed sludge. Fifteen of these seed-MAGs are represented by the co-assembled MAGs (shared average amino acid identity (AAI) > 99.5%). The remaining 9 seed-MAGs were unique in the seed sludge metagenome but only recruited 10.5% reads from the seed sludge, suggesting that the key populations in the seed sludge can be represented by the 49 MAGs from the co-assembled samples. In addition, these 9 seed bacteria were nearly washed out as their MAGs together recruited < 1% of the DNA reads from days 204, 210, 213, and 261. Moreover, the co-assembled 49 MAGs represented the most active populations in the studied PNA reactor, which recruited 86.1% of the transcriptomic reads that could be mapped to all predicted genes of the co-assembled metagenome (Additional file 2: Table S3). In contrast, the 9 MAGs that were only recovered from the seed sludge recruited < 0.5% of the transcriptomic reads. In summary, a total of 58 MAGs were recovered from anammox sludge, which represented the major proportion of the studied microbial community.

Complete or partial 16S rRNA gene sequences were recovered in 18 MAGs, and 15 of them shared 100% gene identity (no gap and mismatch) with amplified 16S rRNA gene sequences (V4 region) (Additional file 2: Table S4). This shows the persistence of the same microbial members from day 1 to day 261. The genome-based shift of the abundant bacteria, such as AMX1, AOB1, PLA1, ARM1, and CFX1, shared similar trends with the community changes from 16S rRNA gene data (Additional file 2: Table S4). However, the relative abundances of several bacteria that were estimated based on shotgun sequencing were different from the amplicon sequencing result, possibly due to the PCR amplification biases [55].

Similar to the amplicon sequence results, a shift in the bacterial community was also found in the studied PNA reactor based on the relative abundance distribution of the newly recovered MAGs (Fig. 2b and Additional file 1: Figure S6). Remarkably, AMX3 accompanied by an assemblage of bacteria was mainly identified in the seed sludge, while it was nearly undetectable in the anammox sludge samples taken after ~ 200 days of SBR operation. In contrast, we found that the low abundant species in the seed sludge, including AOB1 and AMX1, dominated the new bacterial community. In addition to the abundant bacterial populations, 18 MAGs were identified as low relative abundant species (< 0.5%) in both seed and long-term operated anammox sludge samples (Additional file 1: Figure S6).

**Fig. 3:**
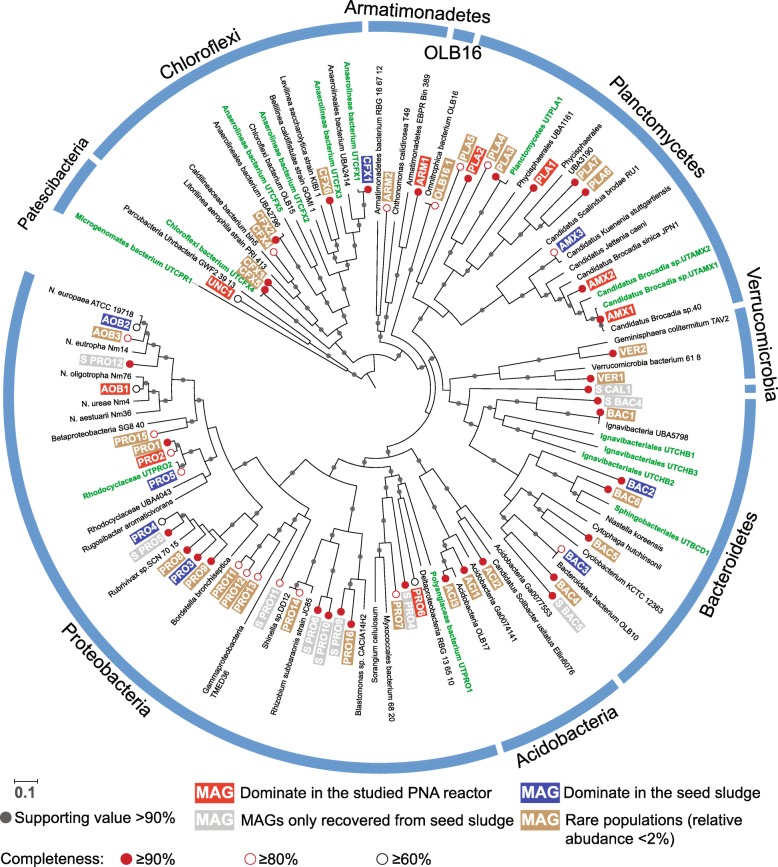
Phylogeny of 120 concatenated proteins from 121 bacterial genomes, including the 58 MAGs from the present study. The identification and alignment of the 120 conserved proteins were performed using the GTDB-Tk [53]. The MAGs recovered from this study are in white. The completeness of these MAGs was estimated using CheckM [40]. MAGs that dominate in the studied PNA reactor (long-term SBR operation) and seed sludge (continuous operation) are in white bold font with red and blue backgrounds, respectively. The unique MAGs that are only recovered from seed sludge are in white bold font with gray backgrounds. Rare populations that have relative abundance < 2% in the seed and long-term operated PNA reactor are in white bold font with brown background. Kartal et al. [19] reported MAGs are in green bold font

As shown in the genome tree (Fig. 3), we recovered three anammox MAGs and three AOB MAGs from anammox sludge. Most of these recovered MAGs (62.0%) were high-quality genomes (> 90% completeness and < 5% contamination) and affiliated with widely reported phyla in anammox systems [56], i.e., *Proteobacteria*, *Bacteroidetes*, *Planctomycetes*, *Chloroflexi*, *Acidobacteria*, *Armatimonadetes*, and *Verrucomicrobia* (Additional file 2: Table S1). The shift in the phylogenetic affiliation of the dominant anammox bacteria in the seed sludge (AMX3, *Candidatus* Kuenenia) and the new stable state (AMX1, *Candidatus* Brocadia) was consistent with the taxonomic shift that was observed from the 16S rRNA gene data.

The most dominant anammox bacterium at the new stable state (AMX1) shared an AAI of 99.1% with *Candidatus* Brocadia sp. 40 [57] and was identical (100%) to *Candidatus* Brocadia sp. R4W10303 [58] (Additional file 2: Table S5). The dominant anammox (AMX3) in the seed sludge shared an AAI of 99.5% with *Candidatus* Kuenenia stuttgartiensis [19]. Several bacteria from the phyla *Chloroflexi* (CFX1 and CFX5) and *Proteobacteria* (PRO5) were closely related to (> 95.5% AAI) the organisms (UTCFX1, UTCFX4, and UTPRO2) that were reported in an anammox reactor by Lawson et al. [20] (Fig. 3)*.* This further confirmed that the anammox-associated organisms can be widely identified in the anammox system. However, the bacteria (UTCFX1) in the previous work [20] that showed a close phylogenetic relationship with the dominant bacteria (CFX1) in the seed sludge of the present study (relative abundance of 34.2%) only had a relative abundance of 1.1%, indicating that the community structure varied greatly along with reactor design and operational strategies.

Although the reactor performance was recovered by changing the feeding strategy, this change in operating conditions radically changed the microbial community, resulting in a shift of key autotrophic organisms and heterotrophic organisms in the PNA reactor. The widely reported shifts in the dominant anammox populations in different studies [59–61] supported that anammox bacteria are likely affected by niche differentiation. van der Star et al. [62] observed a contrary tendency that the anammox population shifted from *Candidatus* Brocadia sp.40 to *Candidatus* Kuenenia stuttgartiensis due to the limiting substrate concentration. In the present study, the seed sludge for the reactor startup was collected from a continuously operated PNA reactor by introducing the synthetic influent at a slow flow rate (7–14 mL/min), which provided limiting substrate concentrations (ammonium and nitrite) in the reactor [29]. This continuous operational mode selected the affinity strategists (AMX3) in the seed sludge. In contrast, the SBR operating mode in the present study created a relatively higher substrate concentration due to the sequencing feeding strategy. Therefore, the SBR operational mode enriched the anammox bacteria that were reported as growth rate strategist [62]. In addition, the observed tiny sludge granules also supported the previous studies that the forms of anammox sludge (e.g., flocs, biofilm, and granules) have an effect on the microbial community assembly in anammox reactors [63, 64]. However, the change in operational mode also changed other parameters such as the aerobic exposure time and sludge retention time (SRT), which may also influence the microbial community assembly [65]. Therefore, further batch investigations will be needed to study how these operational factors change the microbial community in the studied anammox systems. Moreover, assemblages of the dominant heterotrophs shifted in conjunction with the key autotrophs (AOB and anammox bacteria), indicating that biotic interactions may also contribute to the bacterial assembly.

### Nitrogen metabolism as the most active process in the studied PNA reactor

**Fig. 4:**
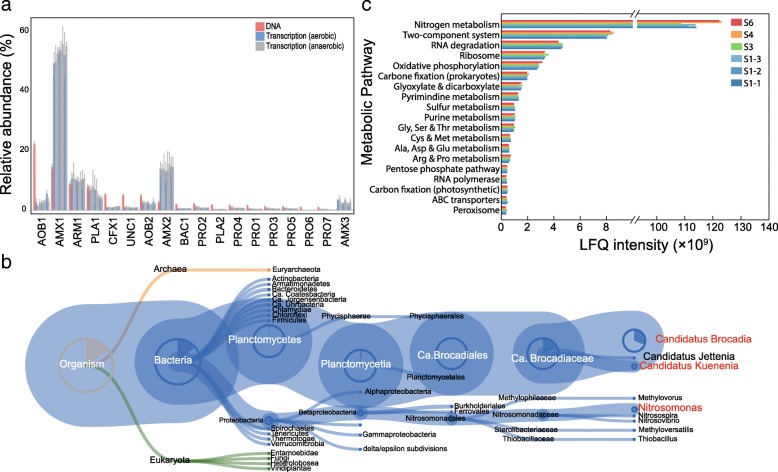
Multi-omic abundance. **a** Relative abundance and time series transcription of the newly recovered MAGs (> 1% DNA-based relative abundance or > 1% transcription-based relative abundance). The relative abundance (normalized by the genome size) and transcription represent the ratios of recruited metagenomic sequences and metatranscriptomic sequences of a given MAG to the total recruited metagenomic and metatranscriptomic sequences of all the recovered MAGs. Triplicate metatranscriptomic data of each sampling point were used for the estimation of transcription in MAGs. The order of relative transcription follows the sampling time series. **b** Taxonomy distribution of identified proteins. Taxa in *Candidatus* Brocadiales and *Nitrosomonadales* are expanded in this tree. **c** Metabolic pathway distribution of the identified proteins; the pathways that have LFQ intensity > 0.35 × 10^9^ are presented

Based on the results of the time-series metatranscriptomes that were obtained from three independent reaction cycles (days 204, 210, and 213), two of the newly recovered anammox bacteria (i.e., AMX1 and AMX2) were the most active bacteria in the PNA reactor under the SBR operational mode, which recruited more than 66% of the transcriptomic reads that could be mapped to all the newly recovered MAGs (Fig. 4a). Consistently, the weighted and unweighted abundances of the identified anammox proteins accounted for 81.1% and 82.9% of the detected proteins in the samples taken from day 261, respectively (Fig. 4b, Additional file 1: Figure S7). Following anammox bacteria, AOB were the second most active bacteria based on the results of protein expression (2.1% weighted abundance and 11.2% unweighted abundance). The proteins for nitrogen metabolism were expressed over 100-fold higher than other metabolic pathways (Fig. 4c and Additional file 1: Figure S8), which demonstrated that nitrogen metabolism was the most important process and may link other heterotrophs with anammox bacteria and AOB.

**Fig. 5:**
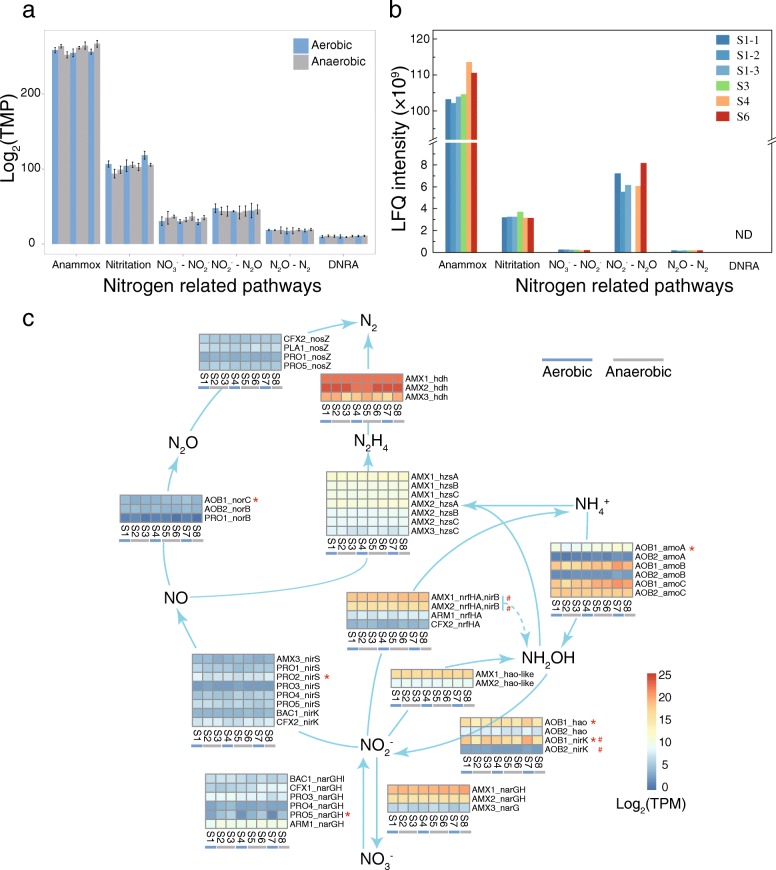
Nitrogen metabolism involved gene and protein expression. **a** The overall transcription of nitrogen metabolism-related pathways. The nitrogen metabolism-related genes in anammox bacteria were individually estimated. The order of transcription value follows the sampling time series. **b** The total LFQ intensity of identified proteins for nitrogen metabolism-related pathways. ND, not detected. **c** Average gene transcription (triplicate samples) of specific gene encoding enzyme for nitrogen metabolism in the time-series anammox sludge samples. The gene affiliation and enzyme names are aligned to the heat maps. The expression profiles that had significantly differential transcriptomic profiles between aerobic and anaerobic samples are marked with red asterisks. The uncertain gene functions are marked with hashtags. The hydrazine synthase subunit A (*hzsA*) and B (*hzsB*) were not recovered in AMX3

Only approximately 1% of all predicted genes (505,204) in the co-assembled metagenomes showed differential transcription across aerobic and anaerobic phases. The genes involved in nitrogen metabolism showed that most genes did not have significantly different transcription patterns across the aerobic and anaerobic phases (Fig. 5). This minimal transcriptomic change confirmed that the transcriptome did not respond to oxygen cycling in the reactor, suggesting that the self-aggregated granules provide niche differentiation for aerobic and anaerobic bacteria [66].

Specifically, the genes for anammox-associated nitrogen metabolism showed the highest transcription and protein expression activities compared with other nitrogen metabolic pathways (Fig. 5a, b). All subunits of the hydrazine synthase (HZS) [19], i.e., *hzsCBA* genes, were identified and actively expressed in AMX1 and AMX2 at both the RNA and protein levels (Additional file 2: Tables S6, S7, and S8 and Additional file 1: Figure S8), and the HZS complex accounted for ~ 80% of all identified proteins for nitrogen metabolism in the anammox bacteria. Genes encoding the three subunits of ammonium monooxygenase (AMO, *amoA*, *amoB*, and *amoC* genes) were actively expressed (log_2_TPM > 10) in the dominant AOB (AOB1). These three genes in AOB were expected to have comparable expression levels, whereas the transcript abundance of the *amoC* genes was ~ 487-fold higher than that of the *amoA* genes in AOB. Notably, the relative gene expression of *amoB* in AOB1 was ~ 500-fold higher than in AOB2 (Additional file 2: Tables S9 and S10), which was confirmed by the metaproteomes (Additional file 1: Figure S8). In addition, one gene encoding copper-containing nitrite reductase (CuNiR) showed extremely high transcription in AOB1 and was significantly upregulated (*P* < 0.01) during the aerobic phases, suggesting that the CuNiR may not solely act as a nitrite reductase in AOB1 (Fig. 5c). A recent study showed that NirK may oxidize NO to nitrite in *Nitrosomonas europaea* [67].

As expected, some of the abundant (> 1% relative abundance) organisms, including CFX1, PLA1, ARM1, BAC1, and PRO2-5, which did not have autotrophic carbon fixation pathways, encoded and transcribed genes for partial or complete denitrification (Fig. 5c) in the studied PNA reactor. The most abundant heterotrophic bacteria (ARM1) encoded and expressed genes for respiratory nitrate reductase (NarGH) and cytochrome c nitrite reductase (NrfH), suggesting that this organism could perform anaerobic respiration using nitrate/nitrite as electron acceptor. Additionally, several other organisms, including the most abundant bacteria in the seed sludge (CFX1), showed the capability of nitrate respiration. Based on the transcription of genes encoding nitric oxide reductase (Nor), nitrous oxide in the studied PNA reactor might be mainly produced by AOB. Four newly recovered bacteria (CFX2, PLA1, PRO1, and PRO5) encoded and expressed nitrous oxide reductase (NosZ), which may reduce the nitrous oxide to nitrogen gas. It is noteworthy that the gene encoding NosZ in one abundant heterotroph, PLA1, was among the top 50 highly expressed genes (Additional file 2: Table S11).

The recovered abundant and/or active heterotrophs in the PNA reactor were denitrifying bacteria and provided extra nitrite/ammonium for anammox bacteria via the nitrite/ammonium loop, which was also identified in previous works [17, 20, 68]. Therefore, the assemblages of bacteria may be selected not only by the operational modes but also by the key metabolism process in the studied system. The transcription for the denitrification process was much lower than that of the anammox and AOB processes in the studied PNA reactor, which may be because no extra carbon source was added in the synthetic influent. Therefore, the changes in key autotrophic bacteria and their metabolic features may also shape the assembly of heterotrophs in the PNA reactor.

### Preference for electron donors in abundant heterotrophs changed in conjunction with the shift in anammox bacteria

The exchange of electron donors is a widely discussed driving force that shapes microbial community assembly [69–71]. As shown in Fig. 5c, the abundant bacteria (> 1% relative abundance) in the studied PNA reactor were mainly denitrifying bacteria that may use nitrite, nitrate, and gaseous nitrogen oxide as electron acceptors to perform anaerobic respiration. In the studied PNA reactor without an additional carbon source, the heterotrophs were anticipated to have intensive interactions with the AOB/anammox bacteria for electron donors.

Genes encoding ABC transporters for carbohydrate and multi-sugars were identified in the most abundant heterotroph (ARM1). Approximately 28% of the 87 identified ABC transporters were predicted to be transporters for carbohydrates in this organism (Additional file 2: Table S12), and at least 145 genes in ARM1 were associated with the currently deposited families from the CAZy database (Additional file 2: Table S13), including 48 genes encoding glycoside hydrolases. In addition, several other organisms enriched by the SBR operational mode (e.g., PLA1 and 2 and PRO6) encoded more glycoside hydrolases genes than the heterotrophic bacteria in the seed sludge.

In contrast, the heterotrophs (CFX1) that dominated in the seed sludge encoded numerous genes for peptidases and peptide transporters (Additional file 2: Table S14). Genes for outer membrane metallopeptidases were identified and highly expressed in CFX1, PRO3, and PRO4 (Additional file 1: Figure S9), suggesting that these organisms were protein degraders in the seed sludge. The key protein degrader (Ignavibacteriales UTCHB1) that was reported by Lawson et al. [20] was not identified in the present study, which further supported the fact that the abundance of the microbial members with similar ecological roles varied greatly in different anammox systems. CFX1 also showed a potential for fatty acid utilization by encoding and transcribing genes for fatty acid degradation. These results revealed that the key organisms in the seed sludge exhibited distinct preferences for carbon sources compared with the key microorganisms after long-term SBR operation.

This distinct preference for electron donors among the heterotrophs could be explained by the shift in dominant autotrophs and the self-aggregation after long-term SBR operation. As the major organic carbon source, EPS are mainly composed of polysaccharides, proteins, nucleic acids, and lipids, which also contribute to the aggregation of bacterial cells [72]. As the most active bacteria in the studied PNA reactor, anammox bacteria may contribute to the majority of the carbon sources. However, the EPS composition of different anammox bacteria varied greatly [59], suggesting that the shift in anammox taxa may induce the change in the major organic carbon sources in the studied PNA reactor. Based on the metatranscriptomes, more transcriptomic reads for amino acid metabolism were observed in AMX3 than in AMX1 and AMX2 (Additional file 1: Figure S10). In contrast, the anammox bacteria that were enriched after the long-term operation showed higher activity with respect to the biosynthesis of polysaccharides. AMX1 (0.8% of transcriptomic reads) showed higher transcriptomic activity for amino sugar biosynthesis than the dominant anammox bacteria in the seed sludge (AMX3, 0.5% of metatranscriptomic reads). Genes encoding alginate export protein were highly transcribed in these three recovered anammox MAGs, but particularly higher in the enriched anammox bacterium (AMX2) after the long-term operation that transcribed > 100-fold higher than its median transcription level in both aerobic and anaerobic phases. Consistently, EPS of one anammox bacterium that shared an AAI of 99.1% with AMX1 was reported to be mainly composed of polysaccharide [59]. In summary, the fraction of polysaccharides and protein in the EPS matrix varied along with the shift in dominant anammox bacteria in the studied PNA reactor, which further shaped the bacterial community with a distinct preference for electron donors.

### Auxotrophies shaped the key bacterial community assembly

**Fig. 6:**
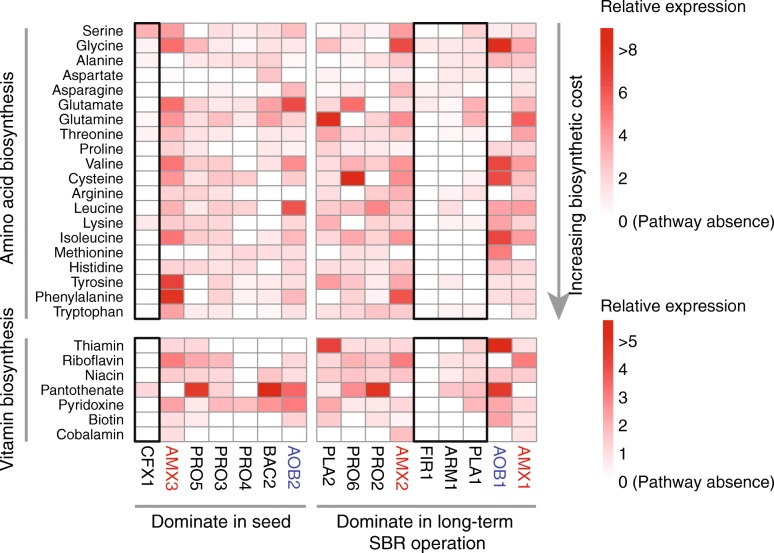
Relative gene transcription of amino acid and vitamin biosynthesis in the abundant bacteria. Transcriptomic expression of pathway was relativized by median TPM values in a given MAG [20]. White box indicates pathway absence (genome-based). The black rectangles highlight the most dominant (> 5% relative abundance) heterotrophs in the seed sludge or the sludge after long-term SBR operation

In addition to electron donors, the exchanges of amino acids, vitamins, and other cofactors of microorganisms were reported to have effects on the microbial community assembly as most microorganisms are auxotrophs [73]. AMX1 and AMX2, identified in the studied PNA reactor, are prototrophic bacteria that can synthesize almost all amino acids, including the amino acids with the greatest metabolic cost, such as tyrosine, phenylalanine, and tryptophan (Fig. 6). Several key genes for alanine, aspartate, and asparagine biosynthesis were not identified in AMX3, which might be due to the relatively low completeness of AMX3 (86.7%). However, genes encoding cystathionine beta-lyase or cystathionine gamma-synthase for methionine biosynthesis were not identified from any anammox MAGs. Interestingly, genes for the methionine biosynthesis process were highly transcribed in AOB1 (~ 5-fold higher than its median level). This result suggested that anammox may also benefit from the metabolism of the amino acids in AOB, although they carry out different metabolic features and compete for substrate (ammonium/ammonia) in the PNA reactor.

It was unexpected that the most abundant heterotrophic bacteria in seed (CFX1) and sludge after long-term SBR operation (PLA1 and ARM1) were auxotrophic for more amino acids than the other relatively low-abundant heterotrophs. Aside from the largely missing genes for amino acid biosynthesis, the transcription of the existing amino acid biosynthesis pathways was generally lower than that of other microbial members (Fig. 6). Based on the reconstructed pathways, CFX1 (14) required more types of extracellular amino acids than PLA1 (9) and ARM1 (8). Consistently, the genes for membrane transportation were one of the most active pathways in CFX1 (6.4% of metatranscriptomic reads) and ARM1 (3.7% of metatranscriptomic reads), supporting that these bacteria rely on other members of the community. Specifically, the genes for L-amino acids, polar amino acids, and branched-chain amino acid transporters were highly transcribed in these two organisms (Additional file 2: Tables S12 and S14). In contrast, the majority of amino acid biosynthesis pathways, including the three amino acids with the greatest metabolic cost, could be identified in these relatively low-abundance bacteria, and some of the amino acid biosynthesis pathways were even highly transcribed (Fig. 6). The relatively higher activity of amino acid metabolism in AMX3 than in the other anammox bacteria might explain the co-occurrence of AMX3 and CFX1 in the seed sludge. After the long-term operation, AMX3 and CFX1 were replaced by AMX1, AMX2, and associated heterotrophic bacteria with fewer auxotrophic genotypes. The further decreasing trend of CFX1 that was observed in the sample taken on day 261 (Additional file 1: Figure S6) confirmed that CFX1 could not adapt to the current condition.

Similarly, genes for the biosynthesis of several B-vitamins were missing in the most dominant heterotrophs. CFX1 possess only one complete pathway for pantothenate (vitamin B_5_) biosynthesis. In addition to CFX1, pantothenate biosynthesis was the most highly expressed vitamin biosynthesis pathway in most of the abundant bacteria. Interestingly, the key genes for (R)-pantoate biosynthesis were missing in the newly recovered anammox MAGs, indicating that the anammox bacteria in the studied PNA reactor might be pantothenate auxotrophs and rely on extracellular pantothenate from other members of the community. Anammox bacteria were the sole organisms that synthesize cobalamin (vitamin B_12_) in the studied core bacterial community in both seed sludge and sludge after long-term SBR operation.

The most abundant heterotrophic bacteria (PLA1) that were enriched after long-term operation might adopt an alternative strategy to thrive in the PNA reactor because the process of membrane transportation was not actively expressed. One significant feature of PLA1 is its numerous genes for secretion systems (SSs). At least 51 genes for SS were found and expressed in PLA1 (Additional file 2: Table S11), including 18, 17, and 16 genes that could be assigned to type II, IV, and VI SSs, respectively. The genes encoding hemolysin-coregulated protein (Hcp), which is a reliable indicator for type VI SSs, were highly transcribed in PLA1. Additionally, genes encoding valine-glycine repeat protein G (VgrG) with a C-terminal extension and PAAR (proline-alanine-alanine-arginine) repeat superfamily, forming a sharp conical extension on the VgrG spike, were identified and transcribed in PLA1. Although the domain of the C-terminal extension of VgrG that might act as effector proteins is still unknown, these active SSs in PLA1 may have a pivotal role in its competition with other bacteria.

The above results of amino acid and vitamin exchange in the studied microbial community provided new insights into the microbial community assembly in the PNA reactor. Conventionally, the auxotrophic genotypes were reported to be selected in nutrient-rich or constant environments [74], but the most dominant heterotrophs in the seed sludge and long-term enriched anammox sludge all lacked much more amino acid and vitamin biosynthetic capabilities than other less abundant heterotrophs. Considering no extra carbon sources in the influent of the anammox reactor, the present study confirmed that the loss of costly amino acid biosynthesis and the highly transcribed membrane transportation process in these most dominant heterotrophs might result in strong fitness over other heterotrophs that were less auxotrophic [75].

On the basis of the Black Queen Hypothesis (BQH), “beneficiaries” that lose essential functions depend on other organisms (that is, “helpers”) for the corresponding metabolites [76]. In the studied PNA reactor, anammox bacteria were the helpers that provided almost all types of amino acids and vitamins to the auxotrophs. The shift in these keystone bacteria and their metabolic activities regarding the costly essential metabolites may change the composition of dominant auxotrophs. The proposed beneficiaries provided certain amino acid or vitamin that might not be synthesized by the helpers, which, in return, acted as helpers in this complicated bacterial community. This might explain why microbes are closely dependent on each other and how auxotrophies shape the bacterial community assembly in the PNA reactor. The significant shift in the most abundant bacterial populations also supported the “storage effects” of low-abundance bacteria. These low-abundance bacteria may be crucial for the response to disturbances [77]. The complex contact-dependent interactions (type VI SSs) further expanded our understanding of the biotic interactions underlying the bacterial community assembly in the PNA reactor.

## Conclusions

Taken together, the integrated multi-omics analysis provided new insights into how environmental conditions and biotic interactions simultaneously shape bacterial community assembly in the ammonium-driven PNA reactor without extra carbon source. These new observations linked the strategies of bacterial community assembly with abiotic (operating mode) and biotic factors (e.g., electron donor exchange, nitrogen metabolism, and auxotrophies) and would be crucial to predict and control the microbial community in anammox processes. However, further studies are still required to determine the genuine role of *amoB* in AOB under oxygen-deficient conditions. In the present study, a bacterial assembly strategy was proposed in a lab-scale PNA reactor feeding synthetic wastewater, and the microbial ecology under other operational strategies requires further work, particularly for the anammox systems treating real wastewater.

## Additional files

Additional file 1: Supplementary information. (PDF 1520 kb)

Additional file 2: Supplementary datasets. (XLSX 1717 kb)

## Data Availability

Raw metagenomic and metatranscriptomic sequences were deposited into the Sequence Read Archive at GenBank under the BioProject accession number PRJNA471375. The mass spectrometry proteomics data have been deposited to the ProteomeXchange Consortium (http://www.proteomexchange.org) via the PRIDE [78] partner repository with the dataset identifiers PXD009787. Scripts used in the present study are available in Github (https://github.com/yulinwang605/Metagenomic-analysis).
