# Supplementary material for: Exploring the effects of operational mode and microbial interactions on bacterial community assembly in a one-stage partial-nitritation anammox reactor using integrated multi-omics
Source: Microbiome. 2019 Aug 28;7:122. doi: 10.1186/s40168-019-0730-6 (PMC6714388; doi:10.1186/s40168-019-0730-6)
Supplement: Supplementary file 1 — Supplementary information. (PDF 1520 kb) [file 40168_2019_730_MOESM1_ESM.pdf]

Supplementary Materials
for

Exploring the effects of operational mode and microbial interactions on bacterial community assembly in a one-stage partial-nitritation anammox reactor using integrated multi-omics

Yulin Wang[1], Qigui Niu[2], Xu Zhang[3], Lei Liu[1], Yubo Wang[1], Yiqiang Chen[1], Mishty Negi[1], Daniel Figeys[3], Yuyou Li[4], Tong Zhang[1]*

[1]Environmental Biotechnology Laboratory, Department of Civil Engineering, The University of Hong Kong, Pokfulam Road, Hong Kong

[2]School of Environmental Science and Engineering, Shandong University, 27# Shanda South Road, Jinan 250100, China

[3]Department of Paediatrics, CHEO Inflammatory Bowel Disease Centre and Research Institute, University of Ottawa, Ottawa, ON, Canada

[4]Department of Civil and Environmental Engineering, Graduate School of Engineering, Tohoku University6-6-06 Aoba, Aramaki, Aoba-ku, Sendai 980-8579, Japan

E-mail zhangt@hku.hk; Tel. 852-28578551; Fax 852-25595337.

This file includes:

Methods

S1 Reactor operation

S2 DNA and RNA extraction and sequencing

S3 Protein extraction, trypsin digestion, and mass spectrometry analysis

S4 Metagenomic and metatranscriptomic analysis

S5 Metaproteomic analysis

S6 Peptidases identification

Figures

Figure S1. Variation of nitrogen components in the influent (ammonium-nitrogen) and effluent (ammonium-nitrogen, nitrite-nitrogen and nitrate-nitrogen) and nitrogen removal rate of the PNA reactor.

Figure S2. Color changes of sludge in the studied one-stage PNA reactor.

Figure S3. Characterization of the anammox granules on day 308. a, Image of the anammox granules. b, Light microscope of the anammox granules. c, Size distribution of the anammox sludge.

Figure S4. Microbial community structures in the anammox sludge samples. The relative abundance of taxonomic groups is estimated based on the sequence percentage of the total 16S rRNA sequences in each activated sludge sample at phylum (a) and genus levels (b). The genera that have relative abundance <1% are assigned to the category of "Others". The taxonomy was presented at the lowest level that can be identified.

Figure S5. Plot of differential coverage binning approach, the corresponding relationships between IDs and MAGs information are listed in Table S1. The coverage of x-axis represents the mapping coverage of metagenomic data originated from the samples taken on days 204, 210 and 213, and the y-axis represents the mapping coverage of metagenomic data originated

from the sample of seed sludge.

Figure S6. Comparison of relative abundance in different anammox sludge samples. The relative abundance (normalized by the genome size) represents the ratio of recruited metagenomic sequences of one given MAG to the total recruited metagenomic sequences of all the recovered MAGs.

Figure S7. Relative abundance (weighted) of bacterial community composition assessed by identified protein. S1-1, S1-2, and S1-3 are the technical repeats of sample S1.

Figure S8. Identified proteins ($\log_{10}$(LFQ intensity)) for nitrogen metabolism in AOB and anammox bacteria. Proteins that could be assigned to specific ORFs of recovered MAGs were represented with MAG ids, the proteins with best hits to the UniProt database were represented with taxonomy (genus level). Differential protein expressions are marked with red asterisk (ratio >1.2 or <0.83 coupled with a P value <0.05).

Figure S9. Relative gene expression of amino acid transporters in CFX1 and identified peptidases in dominant organisms. (a) Relative gene expression of amino acid and peptide transporters in CFX1 throughout the time series, and aligned with the corresponding gene loci. The wiggly lines indicate ends of the contig, and parallel double lines show a break in locus organization. Subfamily types of the encoded transporters are represented by color. (b) ORFs of dominant organisms (relative abundance $>1\%$) were firstly annotated using MEROPS database, and the potential peptidases were further confirmed by CD search. The locations of peptidases were predicted by PSORT, and the extracellular, outer membrane and periplasmic peptidases are shown in this figure. (c) Dynamics of highly expressed genes throughout the time series.

Figure S10. Gene expression profiles of ten selected metabolic pathways in the dominant autotrophs. These were estimated based on the ratios of recruited metatranscriptomic sequences of genes involved in selected pathways to the total recruited metatranscriptomic sequences of the corresponding MAGs.

Methods

S1 Reactor operation

The reactor that was operated in the present study was a modified fermentor (Sartorius BIOSTAT B, Goettingen Germany). At Stage I, the reactor was operated in continuous mode at an HRT of 12 hours, which was well controlled by a computer system equipped with pH, level and DO meters. In order to recover the nitrogen removal rate and avoid the loss of anammox sludge, the operating mode was adjusted to a sequencing batch reactor (SBR) operating mode. The PNA reactor was operated with 8 hours, and a fixed exchange ratio of 50% was employed to achieve an HRT of 16 hours. The 8-hour cycle included 6 min feed phase, 450 min reaction phase (divided into 3 aerated and 3 non-aerated phases), 4 min settling phase [1], 10 min discharge phase and a 10 min idle phase (Fig. 1). The temperature of the reactor was operated at 30°C, and the pH was maintained at between 7.8 and 8.1. The stirrer was set at a speed of 80 rpm to ensure the homogeneous distribution of substrate and biomass and to avoid the disturbance of anammox granules. The reactor was aerated with large air bubbles during the aeration phases to avoid excessive dissolved oxygen, which would result in an oxygen-deficient condition that DO could not be detected in the whole reaction cycle. The reactor was fed with synthetic influent, containing ammonium as the single nitrogen source, mineral salt, and no organic carbon. The ammonium concentration was decreased from 250 mg-N/L to 200 mg-N/L on the 16th day and increased to 250 mg-N/L on the 171st day. The mineral medium in the synthetic influent was made as follows: 40 mg/L KH_2PO_4, 107 mg/L $Na_2HPO_4 \cdot 12H_2O$, 150 mg/L $CaCl_2 \cdot 2H_2O$, 25 mg/L $MgCl_2 \cdot 6H_2O$, and 1 mL/L trace element (8.304 g/L $Na_2 \cdot EDTA \cdot 2H_2O$, 5.000 g/L $FeSO_4 \cdot 7H_2O$, 0.215 g/L $ZnSO_4 \cdot 7H_2O$, 0.120 g/L $CoCl_2 \cdot 6H_2O$, 0.495 g/L $MnCl_2 \cdot 4H_2O$, 0.125 g/L $CuSO_4 \cdot 5H_2O$, 0.110 g/L $Na_2MoO_4 \cdot 2H_2O$, 0.095 g/L $NiCl_2 \cdot 6H_2O$, 0.078 g/L Na_2SeO_3, 0.007 g/L H_3BO_4) [2].

S2 DNA and RNA extraction and sequencing

For the triplicate samples from 3 independent reaction cycles, 1.5 mL of anammox sludge (3500 mg volatile solids /L, VS/L) was used to extract the total RNA with MoBio RNA PowerSoil

total RNA isolation kit (MoBio, USA), resulting in 24 total RNA samples for metatranscriptomic sequencing. The corresponding DNA was then eluted using the RNA PowerSoil DNA elution accessory kit (MoBio, USA), and DNA samples obtained from each reaction cycle (8 samples) were equally mixed based on the DNA concentration, resulting in 3 DNA samples for metagenomic sequencing. Quantities and qualities of genomic DNA and total RNA were checked using the Nanodrop ND 1000 (Thermo Fisher Scientific, USA) and Agilent 2100 Bioanalyzer (Agilent, USA).

For the extracted total RNA samples, the residual genomic DNA was removed using PureLink DNase set (Life Technologies, NY, USA), and the non-rRNA were enriched using Ribo-Zero rRNA Removal Kit for bacteria (Illumina, CA, USA). Reverse transcription was performed using SuperScript II Reverse Transcriptase (Life Technologies, NY, USA) with an initial annealing of random hexamers (Thermo Fisher Scientific, PA, USA), and the complementary DNA (cDNA) was purified with Ampure XP beads (Beckman Coulter, IN, USA), followed by second-strand synthesis. The finally constructed double-stranded cDNA fragments were further processed for library construction.

S3 Protein extraction, trypsin digestion, and mass spectrometry analysis

All samples were concentrated to 7000 mg VS/L for protein extraction. The protein extraction was extracted using the B-PER extraction method. 1.5 mL sludge sample was centrifuged at 15,000 g for 15 min at 4 °C. Discarded the supernatant and resuspended the pellet in 1 mL of B-PER extraction buffer (77 mg of dithiothreitol (DTT), 1 tablet of Complete Mini protease inhibitor, 10 mL of B-PER reagent) [3, 4]. Samples were placed at -80 °C for 1 hr, thawed and incubate for 1 hr on ice. Cells are lysed by bead beating (4 cycles for 40 s at 6 m/s with 2 min breaks on ice) using FastPrep-24™ Homogenizer (MP Biomedicals, California, USA). Samples were centrifuged at 15,000 g for 15 min at 4°C to remove cell debris and the supernatant was transferred to a fresh 2.0 mL tube. The 50:50:0.1 acetone/ethanol/acetic acid was added to the supernatant (1.5 mL) and incubated at 4 °C for overnight to precipitate proteins. Centrifuge the samples at 15,000 g for 20 min at 4 °C to collect the protein pellets. The obtained protein pellets were washed thrice in 200 μL 100% ice-cold acetone and centrifuged at 20,000 g at 4 °C for 20

min every time[5]. Remove the supernatant and freeze-dry (Heto Drywinner 3), the dried samples were stored at -80 °C until LC-MS analysis. For the tryptic digest, 50 μg proteins were reduced and alkylated with 10 mM dithiothreitol and 20 mM iodoacetamide, respectively. One microgram of trypsin (Worthington Biochemical Corp., Lakewood, NJ) was then added to each sample for in-solution trypsin digestion at 37 °C overnight with agitation.

The separation of tryptic peptides was performed on an analytical column (75 μm × 50 cm) packed with reverse phase beads (1.9 μm; 120-Å pore size; Dr. Maisch GmbH, Ammerbuch, Germany) with 4-hour LC gradient from 5 to 35% acetonitrile (v/v) at a flow rate of 200 nl/min. The instrument method consisted of one full MS scan from 300 to 1800 m/z followed by data-dependent MS/MS scan of the 12 most intense ions. A dynamic exclusion repeat count of 2 and repeat exclusion duration of 30 s was used for ion selections. All data were recorded with the Xcalibur software and exported as .raw format for further metaproteomic data analysis.

S4 Metagenomic and metatranscriptomic analysis

The shotgun sequences were quality controlled using CLC Genomics Workbench (v6.04, CLCBio, Qiagen) to get clean reads (average Q value >30) for downstream *de-novo* assembly. The clean reads were co-assembled using CLC's de novo assembly algorithm (CLC Genomics Workbench v6.04, CLCBio, Qiagen) with a *k*-mer of 35 and a minimum scaffold length of 1 Kbp. To get the coverage of scaffolds for genome binning, the metagenomic reads from anammox sludge samples (seed vs integrated data of days 204, 210, and 213) were mapped to co-assembled scaffolds with a similarity fraction of 90% over an 80% read length. Mapping was performed with random nonspecific matches if a read aligned to more than one position with equally good scores. The mapping results were then used to calculate the average coverage of assembled scaffolds. Two-dimensional coverage binning approach was used to retrieve the MAGs of the microbial community member in anammox sludge [6].

After trimming the 15 bp low-quality region at the 3' end, the paired-end metatranscriptomic reads were quality filtered. The high quality (average quality score >30) metatranscriptomic reads were subsequently screened using SortMeRNA v2.1 to remove the rRNA sequences based

on the multiple rRNA databases for bacterial, archaeal and eukaryotic sequences [7, 8]. The non-rRNA reads (ranging from 56 to 73 million) from 24 anammox sludge samples were mapped to all predicted ORFs of the co-assembled contigs using the read mapper of the CLC genomics workbench (v6.04, CLCBio, Qiagen) with a mismatch penalty of 2, an insertion/deletion penalty of 3 and a 95% identity over 90% of the read requirement.

The overall gene expression value of a MAG was estimated based on the proportion of recruited metatranscriptomic reads of all ORFs of given MAG to the all of the reads that mapped to the ORFs of the recovered 49 MAGs. To estimate relative gene expression of a metabolic pathway in recovered MAG, relative gene expressions (relativized by median TPM values across all ORFs within given MAG) of ORFs that involved in given metabolic pathway were averaged by the identified gene number.

S5 Metaproteomic analysis

For the construction of the taxonomy-guided database, protein entries of all the MAGs affiliated genera/families (Supplementary Table S1) were retrieved from UniprotKB database (protein sequences were downloaded at March 11, 2018). In addition, a non-redundant gene catalog predicted genes from the assembled metagenome. The gene catalog was then combined with the retrieved protein sequences from UniprotKB for database search using MetaPro-IQ approach as previously described [5].

Taxonomic and biodiversity analysis of metaproteome data was performed using Unipept [9]. KEGG annotation of the quantified proteins was performed using GhostKOALA [10]. The KEGG annotation of the leading protein (defined as the top rank protein in a group; the ranking is based on the number of peptide sequences, the number of PSMs, and the sequence coverage) in a protein group was used for quantitative analysis. The LFQ intensity of all protein groups annotated with the same KEGG pathway were summed to represent the pathway abundance.

S6 Peptidases identification

The peptidases were identified based on the BLASTP searches against the MEROPS (release 12.0) [11]. The best hits of BLASTP results were filtered and the genes that shared similarity

at >50% with the database were further extracted to be confirmed using the conserved domain search (CD-search) [12], and the genes with peptidase domain regions were finally identified as peptidases.

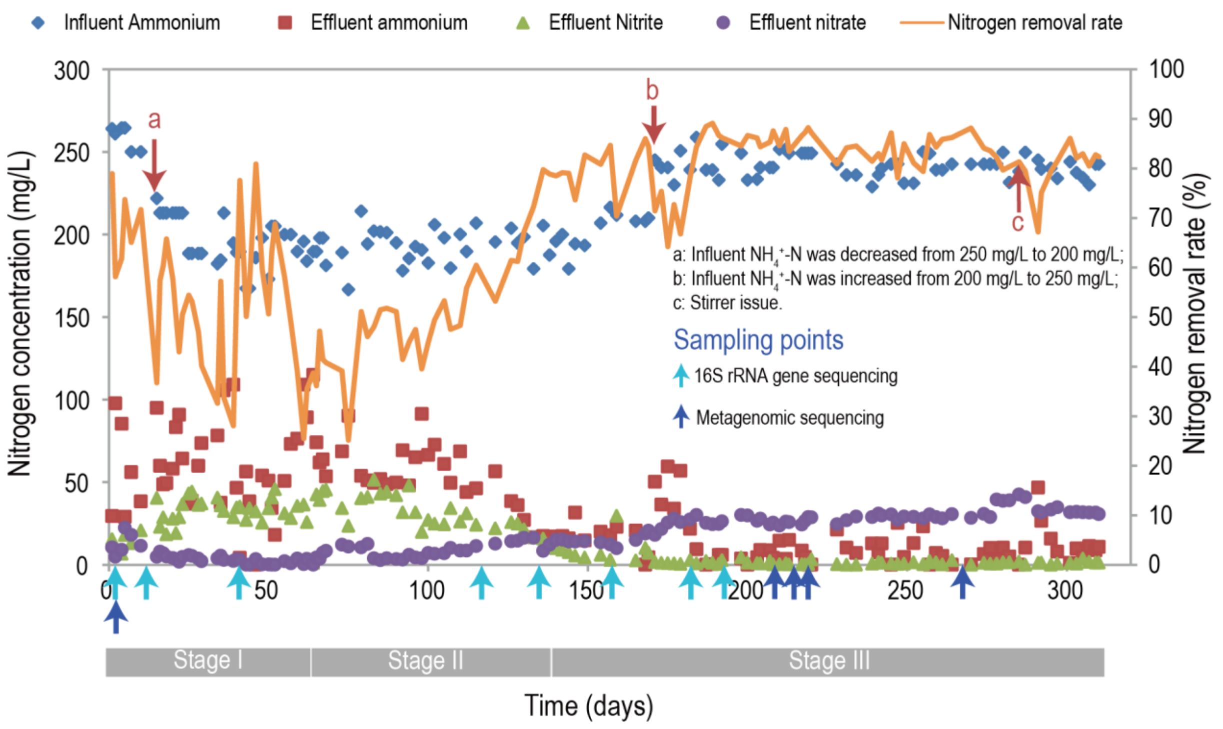

Figure S1. Variation of nitrogen components in the influent (ammonium-nitrogen) and effluent (ammonium-nitrogen, nitrite-nitrogen and nitrate-nitrogen) and nitrogen removal rate of the PNA reactor.

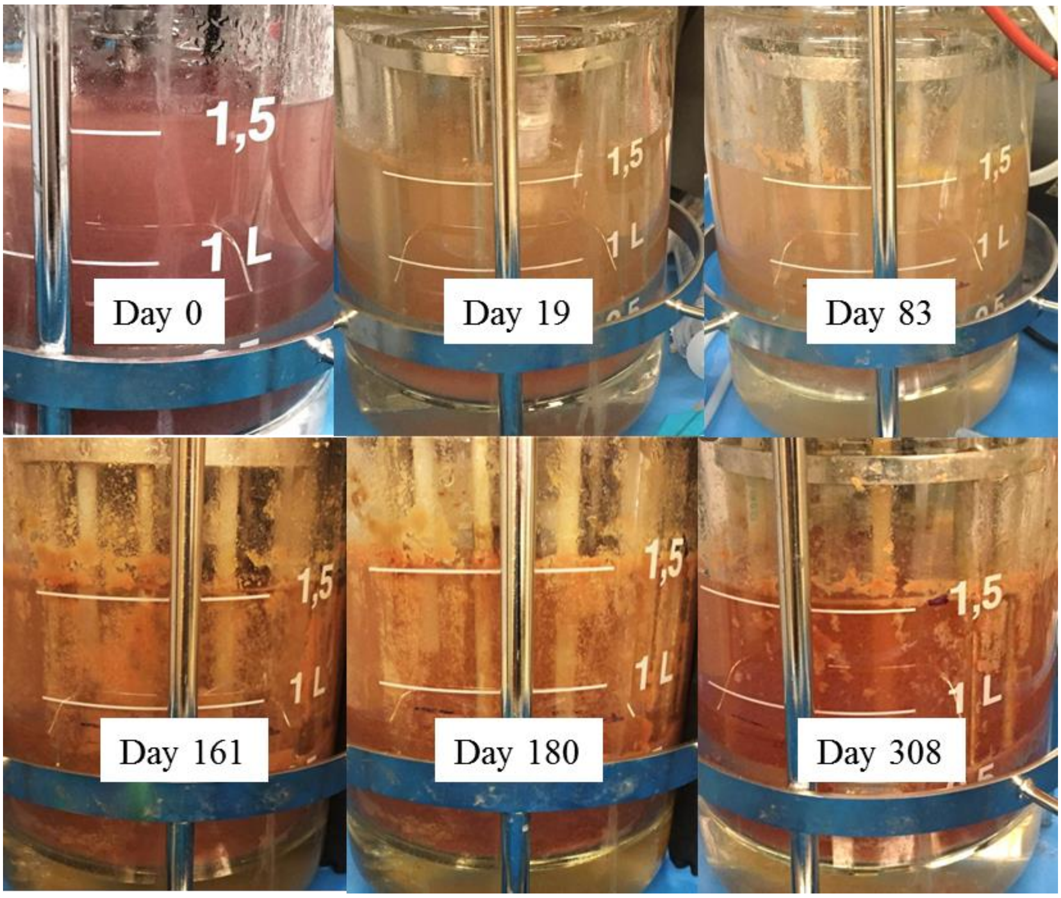

Figure S2. Color changes of sludge in the studied one-stage PNA reactor.

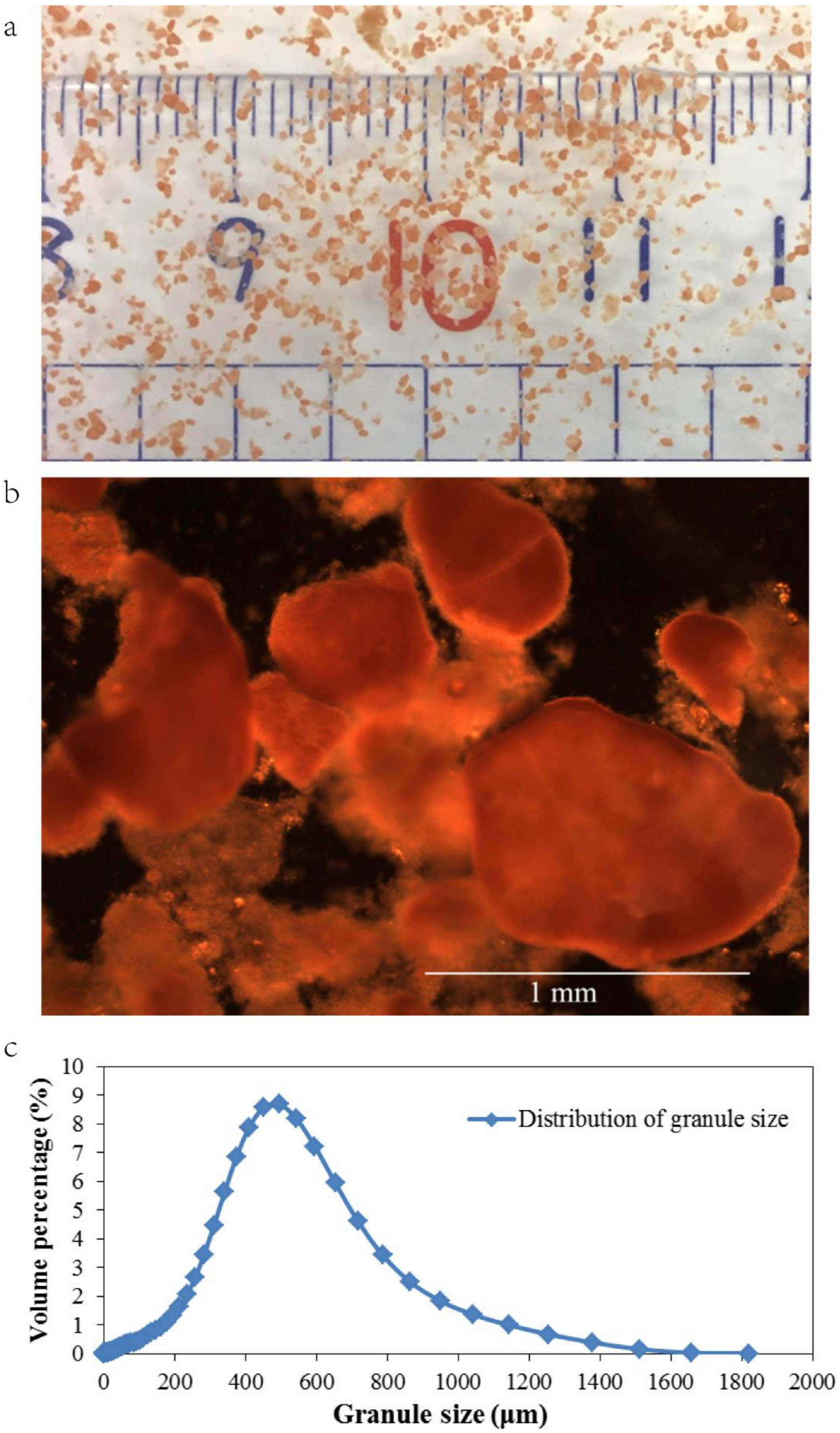

Figure S3. Characterization of the anammox granules on day 308. a, Image of the anammox granules. b, Light microscope of the anammox granules. c, Size distribution of the anammox sludge.

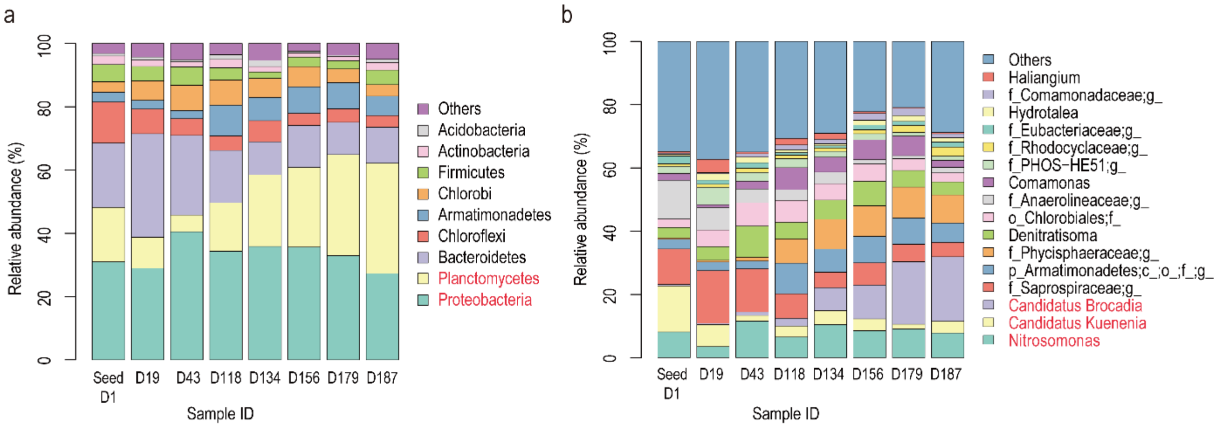

Figure S4. Microbial community structures in the anammox sludge samples. The relative abundance of taxonomic groups is estimated based on the sequence percentage of the total 16S rRNA sequences in each activated sludge sample at phylum (a) and genus levels (b). The genera that have relative abundance <1% are assigned to the category of "Others". The taxonomy was presented at the lowest level that can be identified.

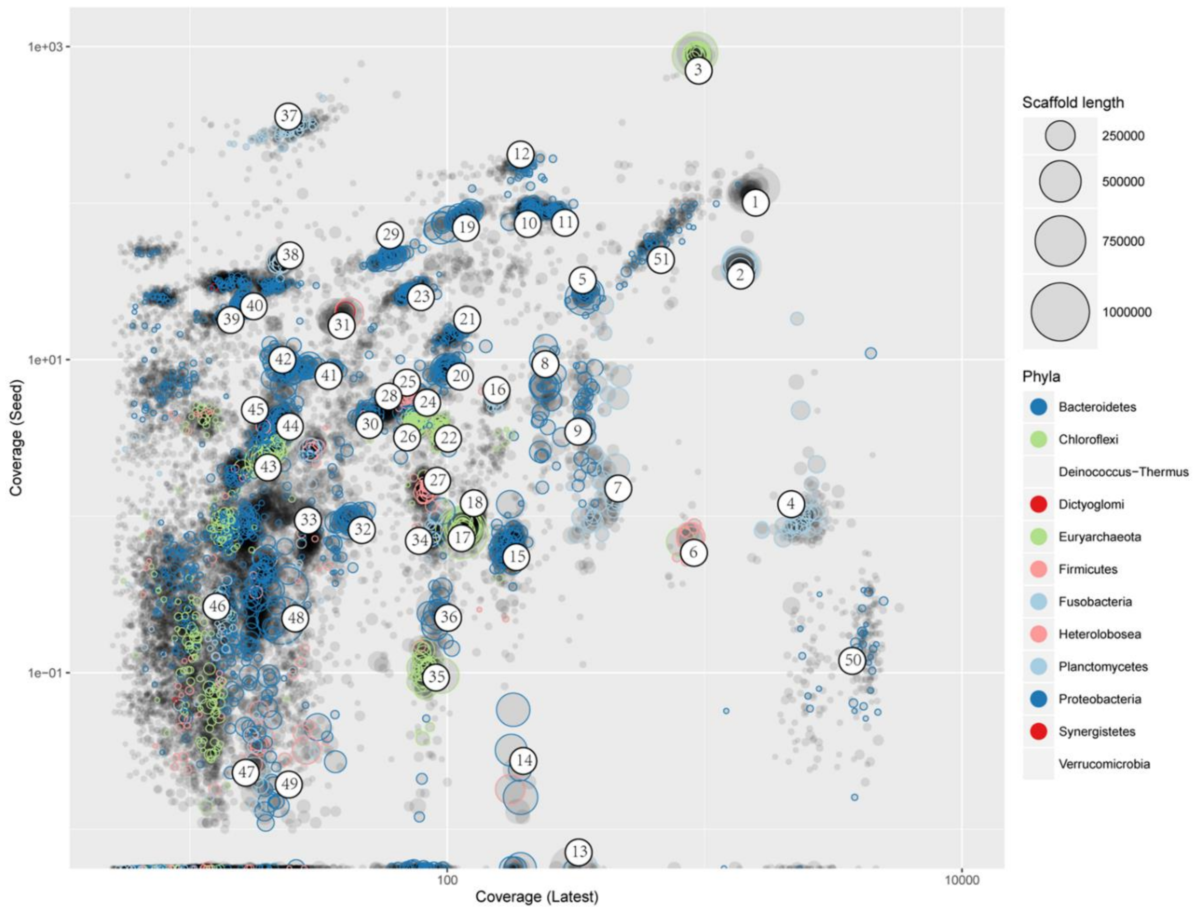

Figure S5. Plot of differential coverage binning approach, the corresponding relationships between IDs and MAGs information are listed in Table S1. The coverage of x-axis represents the mapping coverage of metagenomic data originated from the samples taken on days 204, 210 and 213, and the y-axis represents the mapping coverage of metagenomic data originated from the sample of seed sludge.

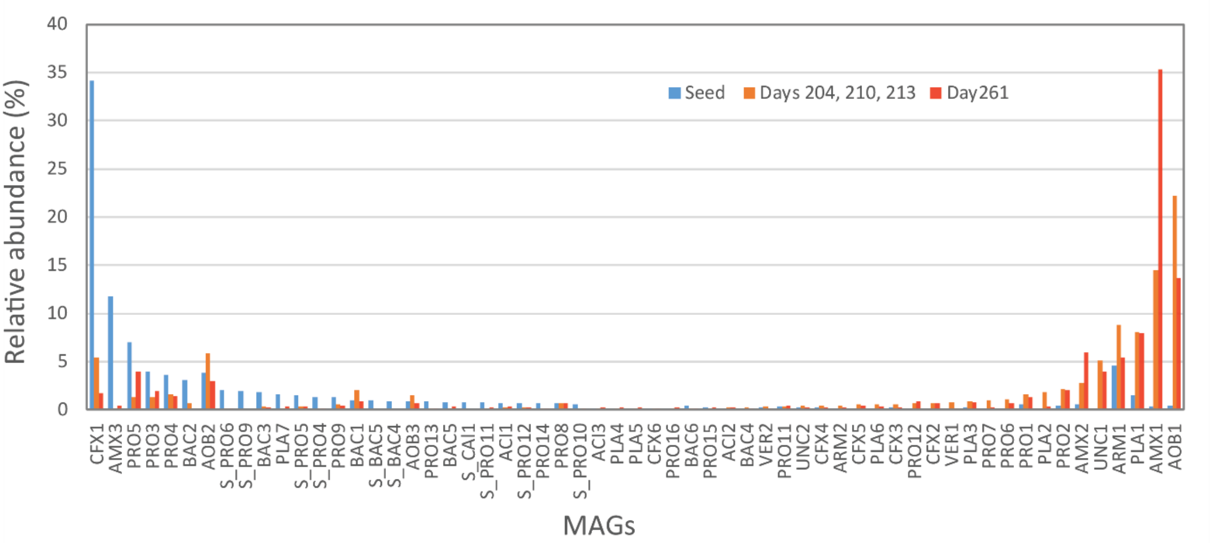

Figure S6. Comparison of relative abundance in different anammox sludge samples. The relative abundance (normalized by the genome size) represents the ratio of recruited metagenomic sequences of one given MAG to the total recruited metagenomic sequences of all the recovered MAGs.

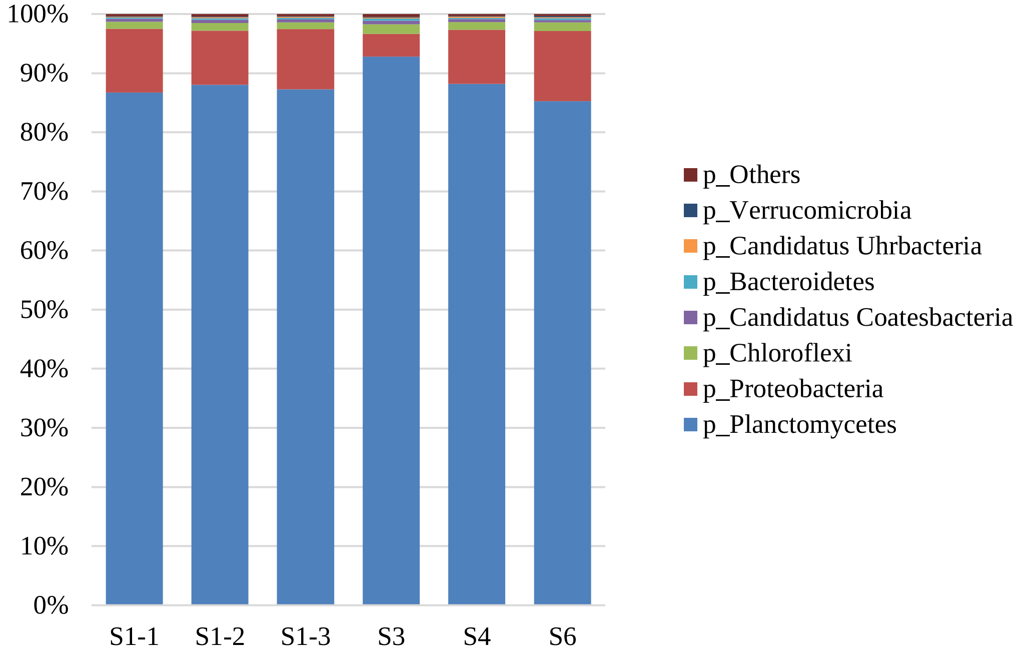

Figure S7. Relative abundance (weighted) of bacterial community composition assessed by identified protein. S1-1, S1-2, and S1-3 are the technical repeats of sample S1.

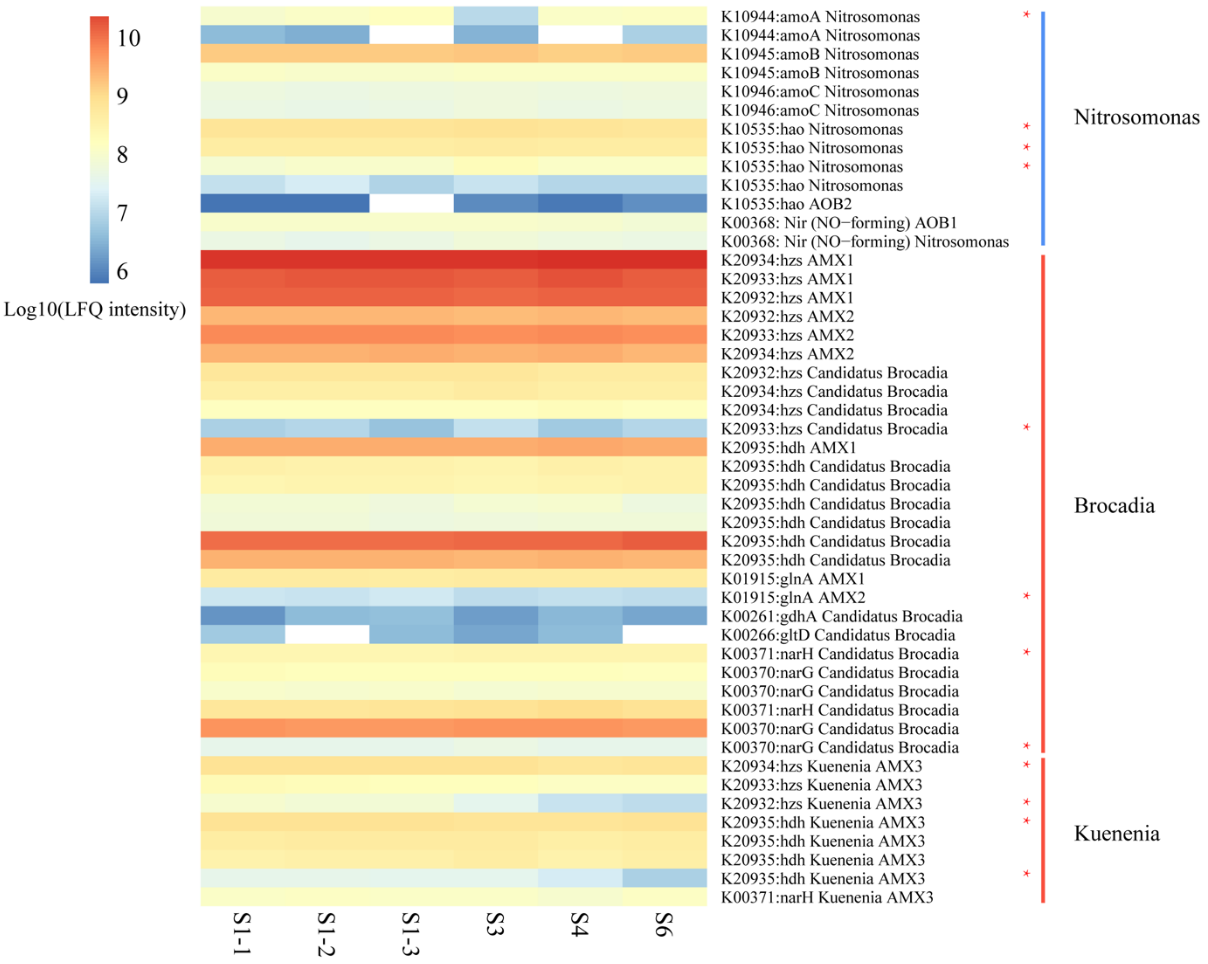

Figure S8. Identified proteins (log10(LFQ intensity)) for nitrogen metabolism in AOB and anammox bacteria. Proteins that could be assigned to specific ORFs of recovered MAGs were represented with MAG ids, the proteins with best hits to the UniProt database were represented with taxonomy (genus level). Differential protein expressions are marked with red asterisk (ratio >1.2 or <0.83 coupled with a P value <0.05).

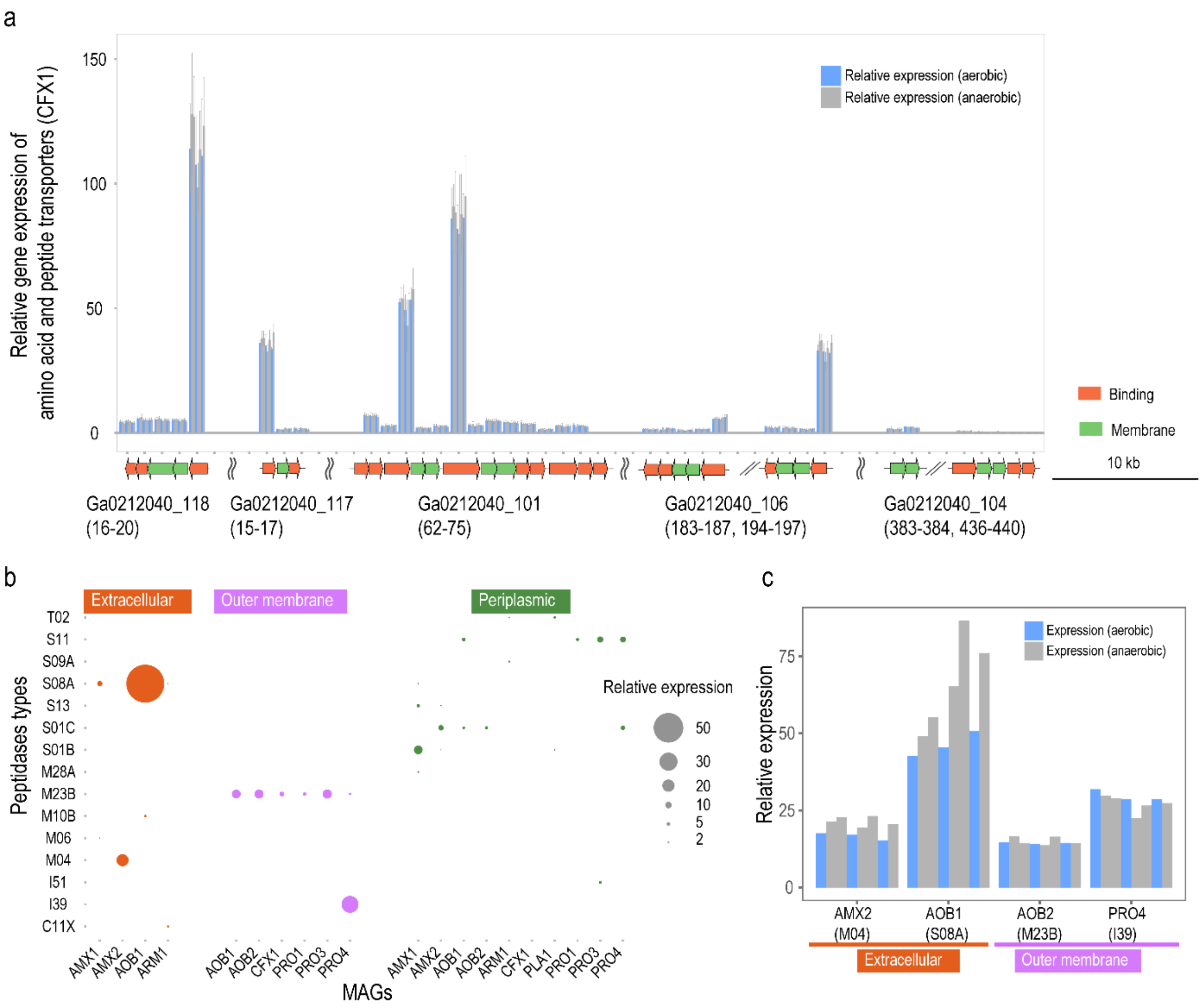

Figure S9. Relative gene expression of amino acid transporters in CFX1 and identified peptidases in dominant organisms. (a) Relative gene expression of amino acid and peptide transporters in CFX1 throughout the time series, and aligned with the corresponding gene loci. The wiggly lines indicate ends of the contig, and parallel double lines show a break in locus organization. Subfamily types of the encoded transporters are represented by color. (b) ORFs of dominant organisms (relative abundance >1%) were firstly annotated using MEROPS database, and the potential peptidases were further confirmed by CD search. The locations of peptidases were predicted by PSORT, and the extracellular, outer membrane and periplasmic peptidases are shown in this figure. (c) Dynamics of highly expressed genes throughout the time series.

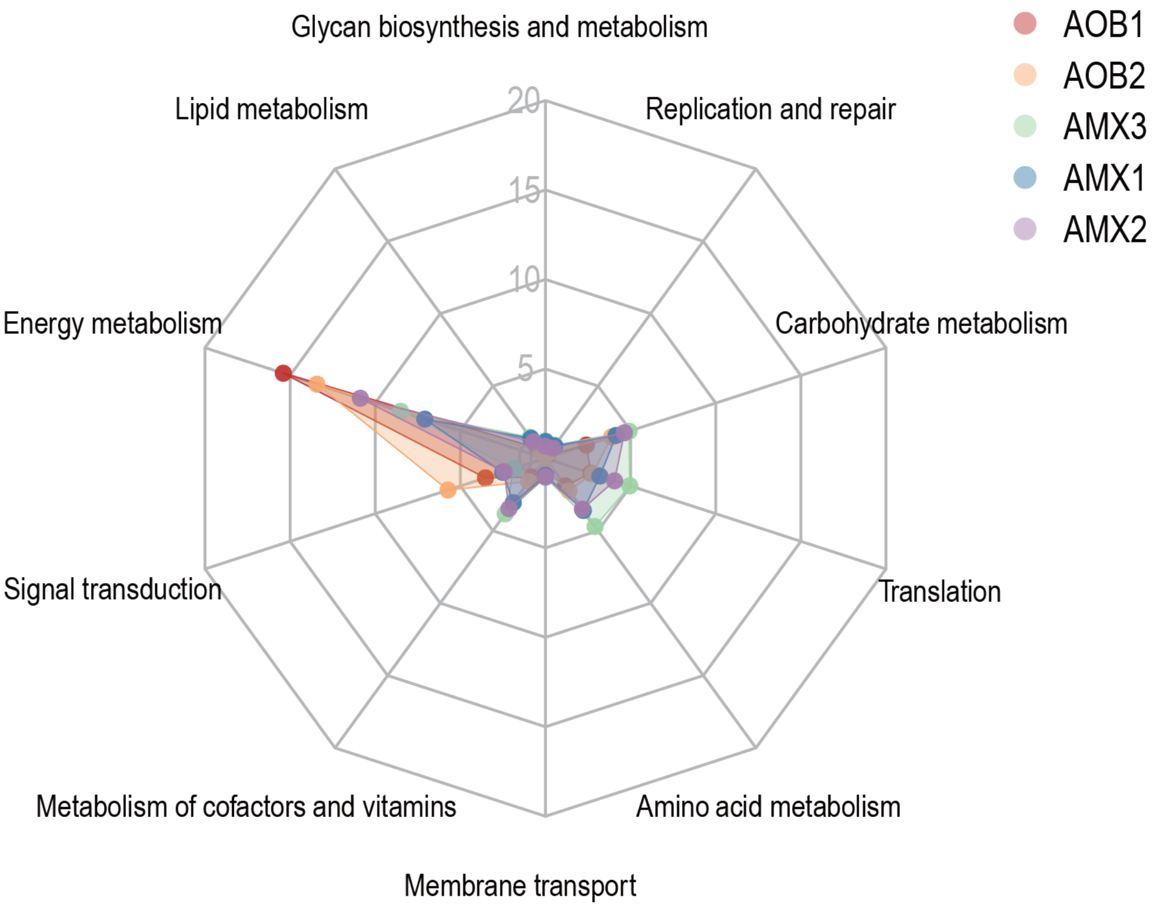

Figure S10. Gene expression profiles of ten selected metabolic pathways in the dominant autotrophs. These were estimated based on the ratios of recruited metatranscriptomic sequences of genes involved in selected pathways to the total recruited metatranscriptomic sequences of the corresponding MAGs.

Reference:

1. Vangsgaard AK. Modeling, experimentation, and control of autotrophic nitrogen removal in granular sludge systems. Unpublished doctoral thesis Technical university of Denmark. 2013.
2. Liu Y, Niu Q, Wang S, Ji J, Zhang Y, Yang M, Hojo T, Li YY. Upgrading of the symbiosis of Nitrosomanas and anammox bacteria in a novel single-stage partial nitritation-anammox system: Nitrogen removal potential and Microbial characterization. Bioresour Technol. 2017;244:463-472.
3. Barr JJ, Dutilh BE, Skennerton CT, Fukushima T, Hastie ML, Gorman JJ, Tyson GW, Bond PL. Metagenomic and metaproteomic analyses of Accumulibacter phosphatis-enriched floccular and granular biofilm. Environ Microbiol. 2016;18:273-287.
4. Hansen SH, Stensballe A, Nielsen PH, Herbst FA. Metaproteomics: Evaluation of protein extraction from activated sludge. Proteomics. 2014;14:2535-2539.
5. Zhang X, Ning Z, Mayne J, Moore JI, Li J, Butcher J, Deeke SA, Chen R, Chiang CK, Wen M, et al. MetaPro-IQ: a universal metaproteomic approach to studying human and mouse gut microbiota. Microbiome. 2016;4:31.
6. Albertsen M, Hugenholtz P, Skarshewski A, Nielsen KL, Tyson GW, Nielsen PH. Genome sequences of rare, uncultured bacteria obtained by differential coverage binning of multiple metagenomes. Nat Biotechnol. 2013;31:533-538.
7. Kopylova E, Noé L, Touzet H. SortMeRNA: fast and accurate filtering of ribosomal RNAs in metatranscriptomic data. Bioinformatics. 2012;28:3211-3217.
8. Lawson CE, Wu S, Bhattacharjee AS, Hamilton JJ, McMahon KD, Goel R, Noguera DR. Metabolic network analysis reveals microbial community interactions in anammox granules. Nat Commun. 2017;8:15416.
9. Mesuere B, Debyser G, Aerts M, Devreese B, Vandamme P, Dawyndt P. The Unipept metaproteomics analysis pipeline. Proteomics. 2015;15:1437-1442.
10. Kanehisa M, Sato Y, Morishima K. BlastKOALA and GhostKOALA: KEGG tools for functional characterization of genome and metagenome sequences. J Mol Biol. 2016;428:726-731.
11. Rawlings ND, Barrett AJ, Finn R. Twenty years of the MEROPS database of proteolytic enzymes, their substrates and inhibitors. Nucleic Acids Res. 2015;44:D343-D350.
12. Marchler-Bauer A, Bryant SH. CD-Search: protein domain annotations on the fly. Nucleic Acids Res. 2004;32:W327-W331.
